# Ready Both to Your and to My Hands: Mapping the Action Space of Others

**DOI:** 10.1371/journal.pone.0017923

**Published:** 2011-04-04

**Authors:** Marcello Costantini, Giorgia Committeri, Corrado Sinigaglia

**Affiliations:** 1 Laboratory of Neuropsychology and Cognitive Neuroscience, Department of Neuroscience and Imaging, University G. d'Annunzio, Chieti-Pescara, Italy; 2 Institute for Advanced Biomedical Technologies, Foundation University G. d'Annunzio, Chieti, Italy; 3 Department of Philosophy, University of Milan, Milan, Italy; University College London, United Kingdom

## Abstract

To date, mutual interaction between action and perception has been investigated mainly by focusing on single individuals. However, we perceive affording objects and acts upon them in a surrounding world inhabited by other perceiving and acting bodies. Thus, the issue arises as to whether our action-oriented object perception might be modulated by the presence of another potential actor. To tackle this issue we used the spatial alignment effect paradigm and systematically examined this effect when a visually presented handled object was located close either to the perceiver or to another individual (a virtual avatar). We found that the spatial alignment effect occurred whenever the object was presented within the reaching space of a potential actor, regardless of whether it was the participant's own or the other's reaching space. These findings show that objects may afford a suitable motor act when they are ready not only to our own hand but also, and most importantly, to the other's hand. Our proposal is that this effect is likely to be due to a mapping of our own and the other's reaching space and we posit that such mapping could play a critical role in joining our own and the other's action.

## Introduction

Several behavioural studies revealed that the mere sight of an object automatically triggers the motor representation of the corresponding action possibilities, in the absence of any effective interaction and even of any intention to act on the object [1], [2], [3], [4]. Indeed, it has been shown that task-irrelevant object information (e.g. the left-right orientation of the handle of a mug) may facilitate the execution of left-right hand motor acts when the orientation of the affording part of the object (e.g. the handle) is spatially aligned with the responding hand [1]. This effect, also called spatial alignment effect, refers to a decrease of reaction times when the subject executes a motor act which is congruent with that afforded by a seen object [5].

Neurophysiological experiments provided these behavioural data with a neuronal counterpart, showing that specific parieto-frontal circuits are devoted to encoding the observed objects in terms of one or more action possibilities both in non human primates [6], [7], [8], [9], [10] and in humans [11], [12], [13], [14].

In a previous study [15] we used the spatial alignment effect paradigm to investigate whether and to what extent the possibility for an object (e.g. a handled mug) to afford a suitable motor act (a hand grasping with a precision grip) might depend on its reachability. We instructed participants to replicate a grasping movement as soon as a task-irrelevant go-signal (i.e. the handled mug placed on a table) appeared. The handle of the mug might elicit a motor representation of a grasping action which is either congruent or incongruent with the grasping action to be executed. Most importantly, the mug could be placed either within or outside the reaching space of the participants. The results showed that the spatial alignment effect occurs only when the task-irrelevant object is presented within the reaching space of the participants.

In everyday life, however, we usually don't perceive and act upon objects by ourselves, because our surrounding world is mostly inhabited also by other perceiving and acting bodies. Therefore the question arises as to whether our action-oriented object perception might be related to and influenced by the presence of other people. To tackle this question we further extended our previous study [15] by introducing a virtual individual such as an avatar in the visual scene and investigating whether the sight of objects located outside the reaching space of the participant but within the reaching space of the avatar might evoke the motor representation of a suitable grasping action as measured by the spatial alignment effect.

As in the previous study, we instructed participants to replicate a seen grip by performing a reach-to-grasp motor act, with either their right or their left hand, on presentation of a task-irrelevant go signal depicting a 3D scene with a mug placed on a table, with its handle oriented towards the right or the left (i.e., congruent or not with the movements to be executed). The mug could be located either within or outside the reaching space of the participants. Differently from the previous study, however, in half of the trials an avatar was seated at the table. The results not only corroborated our previous findings, showing that the spatial alignment effect occurs only when the object falls within the reaching space of the participants, but extended them, demonstrating that the spatial alignment effect occurs even when the mug is presented outside the reaching space of the participants but within the reaching space of the avatar.

## Experiment 1

### Methods

#### Participants

20 healthy subjects took part in this experiment (12 females, mean age 25 y, range 22–28). All subjects were right-handed, had normal or corrected-to-normal visual acuity, were naive as to the purposes of the experiment and gave their written informed consent. The study was approved by the Ethics Committee of the “G. d'Annunzio” University, Chieti, and was conducted in accordance with the ethical standards of the 1964 Declaration of Helsinki.

#### Stimuli, task and procedure

**Figure 1:**
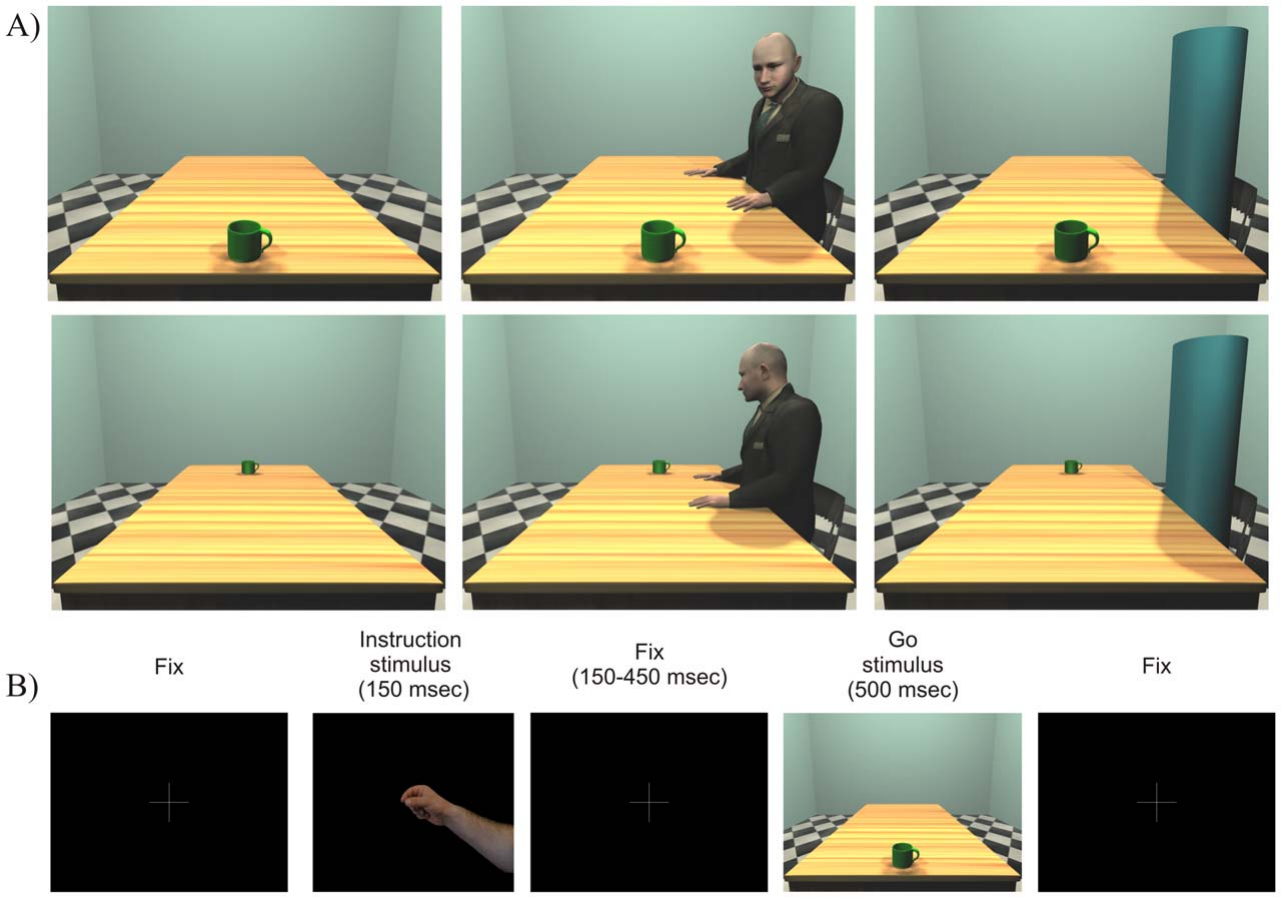
Exemplar go stimuli for Experiment 1 (Panel A). B depicts an exemplar trial from Experiment 1.

Two sets of stimuli were used. The first set of stimuli included coloured pictures depicting either a right or a left hand pantomiming a precision grip movement (instruction stimuli). The second set of stimuli included 3D scenes (Go-stimuli). The scenes were 3D rooms, with a table and a mug on it, created by means of 3D Studiomax v.13. The handle of the mug could be oriented toward left or right (see Fig. 1, Panel A). In half of the trials the mug was placed within the near peripersonal space (30 cm) of the participants while in the other half it was in the far extrapersonal space (150 cm). Moreover, within each spatial sector, in one third of the trials an avatar was seated on a chair on the long side of the table, facing the object, while in another third of the trials a non-corporeal object, namely a cylinder, was placed on the same chair. It is important to note here that both the avatar and the cylinder occupied the same area. When either the avatar or the cylinder was present it was seated on the same side of the table as that toward which the handle was directed, thus being placed either on the right or on the left side of the table.

Thus, the experimental design was a 3×2×2 factorial. The three factors were (i) the Position of the Mug with respect to the participant (Reachable Vs Non-Reachable); (ii) the Position of the Handle with respect to the hand the participants had to use to replicate the grasping movement (Congruent Vs Incongruent); (iii) the Presence of a virtual individual such an avatar (Absent Vs Cylinder Vs Avatar).

Stimuli were presented on a 17′ computer display. Each trial consisted of the presentation of the instruction stimulus for 150 ms followed, after a variable delay (ranging from 150 to 450 ms), by the go stimulus lasting 500 ms. Participants were requested to replicate the reach-to-grasp motor act, including the grip, presented in the first set of stimuli (instruction stimuli) as soon as the go stimulus appeared on the computer display (see Fig. 1, Panel B). Thus, congruent trials refer to the condition in which a participant had to replicate a grasping movement with either the right or the left hand and the handle was located ipsilaterally, while incongruent trials refer to the condition in which the responding hand and the handle were in opposite hemispaces. At the beginning of each trial, participants rested their index fingers on two response buttons arranged horizontally on a button box. Responses were given by lifting the index finger of the response hand and then making the grasping movement as instructed. This allowed us to measure liftoff time (i.e., the time between onset of the go-stimulus and initial hand movement). Each participant provided us with 16 trials per condition. The presentation of the stimuli and the recording of the participants' responses (in terms of movement onset) were controlled by a custom software (developed by Gaspare Galati at the Department of Psychology, Sapienza Università di Roma, Italy; [16]), implemented in MATLAB (The MathWorks Inc., Natick, MA, USA) using Cogent 2000 (developed at FIL and ICN, UCL, London, UK) and Cogent Graphics (developed by John Romaya at the LON, Wellcome Department of Imaging Neuroscience, UCL, London, UK). At the end of the experiment participants were requested to judge the distance of the objects with respect to their bodies. Near and Far stimuli were judged as being 40 cm (SD = 15) and 140 cm (SD = 10) far away, respectively. The judgement was not influenced by the presence of the avatar and the cylinder.

### Results

Trials in which subjects failed to respond (1.2%) were discarded from the analysis. The mean RT of the correct responses was calculated for each condition; responses longer than 2 standard deviations from the individual mean were treated as outliers and not considered (3%). Data were entered in a tree-way ANOVA with: Mug Position (MP, Reachable Vs Non-Reachable), the Handle Position (HP, Congruent Vs Incongruent) and the Presence of another Individual (PaI, Absent Vs Cylinder Vs Avatar) as main factors.

**Figure 2:**
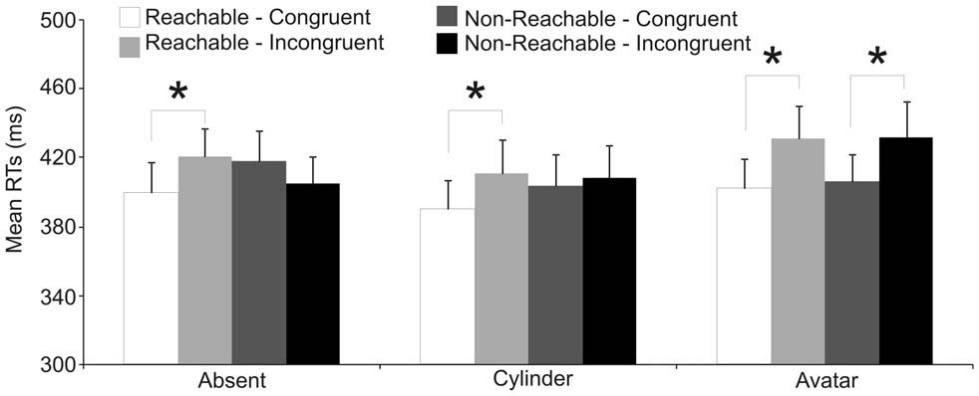
Mean reaction times in Experiment 1. Error bars indicate standard errors.

RT analysis revealed the main effect of Handle Position (HP: F(1,19) = 7.17, p<0.05, η_p_^2^ = .27) with RTs to congruent trials (403.3 ms) faster than RTs to incongruent trials (417.8 ms). HP significantly interacted with Mug Position (HP by MP interaction: F(1,19) = 5.6, p<0.05, η_p_^2^ = .23) given that faster RTs to congruent (397.4 ms) than incongruent trials (420.6 ms, p<0.01) were observed only in the Reachable space (see Fig. 2). This confirms our previous finding of a spatial alignment effect occurring only when the object is within the participant's reaching space [15].

**Figure 3:**
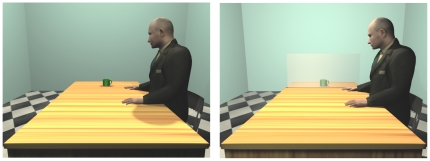
Exemplar go stimuli for Experiment 2.

Crucially for the present investigation, HP and MP significantly interacted with the Presence of another Individual factor (HP by MP by PaI interaction: F(2,38) = 3.3, p<0.05, η_p_^2^ = .15). Post-hoc analysis showed that when another individual was present on the scene (Avatar condition) the above-reported HP effect in terms of congruency gain was observed both within reachable and non-reachable space (Reachable: 402.0 Vs 430.9 p<0.01; Non-Reachable: 406.1 Vs 431.7, p<0.01). In the Absent and Cylinder conditions, instead, the congruency effect was restricted to the reachable space (Absent condition: 399.8 Vs 420.7, p<0.05; Cylinder condition: 390.5 Vs 410.7; p<0.05, see Fig. 3), in line with the HP by MP interaction. It is worth reminding here that when the mug was presented outside the reaching space of the participants it fell within the reachable space of the avatar.

## Experiment 2

In the previous experiment we found that the mere presence of the avatar impacted on the spatial alignment effect even when the mug was outside the reaching space of the participants. However, one may argue that such an effect could be a mere by-product of joint attention phenomena. To the latter regard, it has been shown that simply observing an actor looking at an object does recruit the sensory-motor system of the onlooker [17], [18].

Thus, the fact that in our experiment the avatar always faced the object could be construed as a both necessary and sufficient condition for the recruitment of the participant's motor representation relative to the affording feature of the presented object. To disentangle this question we ran a second experiment in which we interposed a near-transparent panel between the avatar and the affording object. This panel did not prevent the avatar from seeing the object, but did prevent the possibility to interact with it. This manipulation allowed us to assess whether the spatial alignment effect was due to unspecific attentional cues or to the actual reachability of the affording object.

### Methods

#### Participants

20 healthy subjects took part in this experiment (9 females, mean age 25 y, range 23–28). All subjects were right-handed, had normal or corrected-to-normal visual acuity, were naive as to the purposes of the experiment and gave their written informed consent. The study was approved by the Ethics Committee of the “G. d'Annunzio” University, Chieti, and was conducted in accordance with the ethical standards of the 1964 Declaration of Helsinki.

#### Stimuli, task and procedure

In this experiment, the mug was always placed within the near peripersonal space of the avatar and in the far extrapersonal space of the participants. However, differently from experiment 1, in half of the trials the mug was located beyond a Plexiglas panel (Non-Reachable sector of the avatar's peripersonal space). In the other half it was located in front of the same panel (Reachable sector of the avatar's peripersonal space, see Fig. 3). The task and the procedure was the same as in the previous experiment. Thus, the experimental design was a 2×2 factorial. The two factors were (i) the Position of the Mug with respect to the avatar (Reachable sector of the avatar's peripersonal space Vs Non-Reachable sector of the avatar's peripersonal space), (ii) the Position of the Handle (Congruent Vs Incongruent) with respect to the hand participants had to use to replicate the grasping movement.

### Results

**Figure 4:**
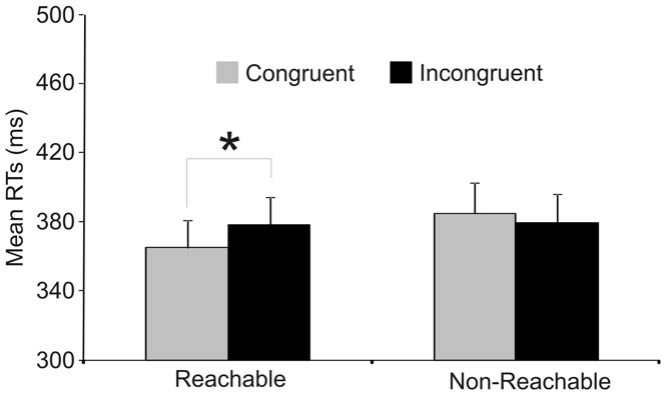
Mean reaction times in Experiment 2. Error bars indicate standard errors.

Trials in which subjects failed to respond (1.1%) were discarded from the analysis. The mean RT of the correct responses was calculated for each condition; responses longer than 2 standard deviations from the individual mean were treated as outliers and not considered (1.5%). Data were entered in a two-way ANOVA with Mug Position (MP, Reachable sector of the avatar's peripersonal space Vs Non-Reachable sector of the avatar's peripersonal space), and Handle Position (HP, Congruent Vs Incongruent) as within-subject factors. RT analysis revealed the main effect of Handle Position (F(1,19) = 12.2, p<0.01, η_p_^2^ = .39) with RTs to congruent trials (371.7 ms) faster than RTs to incongruent trials (382.3 ms). Interestingly, the interaction between Handle Position and Mug Position was significant (F(1,19) = 8.7, p<0.01, η_p_^2^ = .31). The HP by MP interaction was explained by the fact that faster RTs to congruent (365.0 ms) than incongruent trials (385.0 ms, p<0.01) were observed only when the mug was in the Reachable sector of the avatar's peripersonal space (see Fig. 4). In other words, the HP effect in terms of congruency gain did not emerge when the mug was merely near the potential co-actor, but only when it was reachable by him.

## Discussion

In this study we aimed to investigate whether and to what extent our perception of the affording features of an object may be related to and influenced by the presence of another individual. To this purpose we took advantage of the spatial alignment effect paradigm, referring to a decrease of reaction times when a subject executes a motor act which is congruent with that afforded by a seen object [5]. In a previous study [15] we demonstrated that such an effect is space-constrained (see also: [19]), occurring only when the affording object (e.g. a handled mug) falls within the reachable space of the participants. In the present study we further expanded this result by showing that the spatial alignment effect might occur also when the affording object is located outside the reachable space of the participants, provided that it is located within the reachable space of another individual, such as an avatar. Crucially, no spatial alignment effect was observed when the affording object was far from the participants but close to a non corporeal object, such as a cylinder.

One might argue that the presence of someone else on the scene facing the object (or the fact that the avatar has bodily parts extending towards the mug while the cylinder doesn't) could be enough, per se, to prime the motor system of the participants to react more quickly. The presence of an individual gazing at the object has been demonstrated to be a necessary condition for the recruitment of the onlooker's sensory-motor system [17], [18], and this is also the case for the present study. However, the gaze-object relation cannot be considered a sufficient condition for the spatial alignment effect. Indeed, in the second experiment we introduced a near transparent barrier dividing the visual space of the avatar in both a visual reachable and a visual non-reachable sub-space. We found that the spatial alignment effect occurred only when the affording object was actually reachable by the avatar and not only faced by it, that is, when the object was literally ready-at-hand. This clearly indicates that the gaze-object relation is not sufficient per se for the alignment effect to occur.

Overall, our findings indicate that the visual features of an object may suggest or even demand a motor behaviour to the observer not only when the object is located within her own reaching space, but also when it falls within the reaching space of another individual. Our proposal is that such an effect is likely to be due to a mapping of one's own and others' arm reaching space. This does not imply that participants actually extend their own reaching space, thus encompassing the space around the avatar. Rather, they map what is ready to the avatar's hand as if it was ready to their own hand. As a consequence, the seen object might afford a given action either *directly*, when it falls within the participants' own reaching space, or *indirectly*, when it falls within the avatar's reaching space. This seems to be consistent also with the fact that both in the cylinder condition and in the avatar with barrier condition the presented object did not evoke any motor representation in the participants because the scene prevented any actual object-related interaction, being the presented object either close to a non corporeal object or out of reach from the avatar's arm, respectively.

Although the interpretation we propose warrants to be further corroborated, there is evidence that the other's bodily space might be mapped onto one's own body representation. Earlier neuropsychological [20] and behavioural [21], [22] studies showed that a visuo-tactile mapping can be found in humans at the level of bodily (or personal) space. More recently, Thomas and colleagues [23] used a cueing paradigm to investigate the putative role of this spatial mapping in the processing of sensory events on one's own body or on others' body. Cues consisted in brief flashes of light at one of several locations on the other's body, while the target was a tactile stimulus delivered either at the same anatomical location on the participant's body as the preceding visual cue on the model (congruent) or at a different location (incongruent). The results showed a significant congruency effect for anatomical body position, as participants were faster at detecting tactile stimuli on their own body when a visual stimulus was delivered at the same location on the body of another individual. Crucially, this effect was body-specific, not occurring when visual cues were delivered at a non-bodily object (e.g. an house). According to the authors, these findings suggest that the visual-tactile mechanism critical for mapping one's own bodily space might also be used for mapping the bodily space of others, thus providing an interpersonal bodily space representation [23].

The putative neuronal counterpart of such an interpersonal bodily space representation has been provided by Ishida et al. [24]. They recorded bimodal neurons from the ventral intraparietal area (VIP) of the macaque brain. Most of the recorded neurons exhibited visual receptive fields in register with the tactile ones and anchored on a single bodily part (face, forearm, hand, trunk, leg, etc.), selectively responding to the visual stimuli delivered within the peripersonal space of the monkey. However, a significant portion of VIP bimodal neurons exhibited both visuo-tactile RFs on the monkey's body and visual RFs close to the experimenter's body, selectively discharging when a visual stimulus was delivered at 120 cm from the monkey's bodily parts but close to the corresponding experimenter's bodily parts. When visual stimuli were presented at the same distance from the monkey but in the absence of the experimenter, the responses were almost absent. The authors suggested that these neurons might contribute to the spatial mapping of one's own and of the other's bodies [24], [25].

Our data are in line with these findings and suggest to extend the bodily space mapping from the visuo-tactile to the motor domain. Indeed, they show that the mapping of one's own and others' bodily spaces may occur also at the level of the arm reaching space. Interestingly, this reaching space mapping would enable one not only to localize the sensory stimuli around the body of others, but also (and above all) to grasp their body as a situated body which might be afforded by the surrounding things, provided that the latter are ready to hand.

The last issue that needs to be addressed is whether one could really map the reaching space of a virtual individual as an avatar. There is no doubt that our experimental setup differs from a real situation, all the more because the avatar was always presented in the same static posture. However, stimuli similar to those employed in this study have been successfully used to investigate high-level phenomena such as, for instance, explicit perspective taking (e.g. [26], [27], [28]). In particular, in the works by Amorim [26] and Lambrey et al. [27], the visual scenes were created with the same software as our own and presented with the same technology, and the mere presence of a static avatar was able to prime the future viewpoint on the scene. To this regard, it is worth noting that in the present study the avatar's presence on the scene was task-irrelevant. Although we cannot exclude relevant differences between a real person and a computer-generated avatar, in our experimental setup the object-avatar relation was enough to suggest or even demand a motor behaviour to the observer, provided that the object fell within the arm reaching space of the virtual actor.

Let us conclude by recalling what Maurice Merleau-Ponty writes in the Phenomenology of Perception (1962; p. 100), where he claims: “[…] my body appears to me as an attitude directed towards a certain existing or possible task. And indeed its spatiality is not, like that of external objects […], a spatiality of position, but a spatiality of situation.” Here we propose to enrich this view of the body and its spatiality, referring to the way in which we map our own and others' body as potentially acting upon the surrounding objects. We believe that this mapping, though requiring further investigations, might play a relevant role in highlighting not only how individuals perceive affording objects but also how they become able to jointly act upon them [29].
